# Immune genes and divergent antimicrobial peptides in flies of the subgenus Drosophila

**DOI:** 10.1186/s12862-016-0805-y

**Published:** 2016-10-24

**Authors:** Mark A. Hanson, Phineas T. Hamilton, Steve J. Perlman

**Affiliations:** 1 Department of Biology, University of Victoria, Victoria, BC Canada; 2 Integrated Microbial Biodiversity Program, Canadian Institute for Advanced Research, Toronto, Ontario Canada

**Keywords:** Diptericin, Drosocin, *Drosophila*, Immunity, Antimicrobial peptide, AMP

## Abstract

**Background:**

*Drosophila* is an important model for studying the evolution of animal immunity, due to the powerful genetic tools developed for *D. melanogaster*. However, *Drosophila* is an incredibly speciose lineage with a wide range of ecologies, natural histories, and diverse natural enemies. Surprisingly little functional work has been done on immune systems of species other than *D. melanogaster*. In this study, we examine the evolution of immune genes in the speciose subgenus Drosophila, which diverged from the subgenus Sophophora (that includes *D. melanogaster*) approximately 25–40 Mya. We focus on *D. neotestacea*, a woodland species used to study interactions between insects and parasitic nematodes, and combine recent transcriptomic data with infection experiments to elucidate aspects of host immunity.

**Results:**

We found that the vast majority of genes involved in the *D. melanogaster* immune response are conserved in *D. neotestacea*, with a few interesting exceptions, particularly in antimicrobial peptides (AMPs); until recently, AMPs were not thought to evolve rapidly in *Drosophila*. Unexpectedly, we found a distinct diptericin in subgenus Drosophila flies that appears to have evolved under diversifying (positive) selection. We also describe the presence of the AMP drosocin, which was previously thought to be restricted to the subgenus Sophophora, in the subgenus Drosophila. We challenged two subgenus Drosophila species, *D. neotestacea* and *D. virilis* with bacterial and fungal pathogens and quantified AMP expression.

**Conclusions:**

While diptericin in *D. virilis* was induced by exposure to gram-negative bacteria, it was not induced in *D. neotestacea*, showing that conservation of immune genes does not necessarily imply conservation of the realized immune response. Our study lends support to the idea that invertebrate AMPs evolve rapidly, and that *Drosophila* harbor a diverse repertoire of AMPs with potentially important functional consequences.

**Electronic supplementary material:**

The online version of this article (doi:10.1186/s12862-016-0805-y) contains supplementary material, which is available to authorized users.

## Background

The ability to defend oneself from parasites and pathogens (natural enemies) is essential for life, and animals have conserved sophisticated mechanisms of defence referred to as the innate immune system. The innate immune response requires recognition, signaling, and activation of defensive mechanisms. This defence response culminates in the synthesis and secretion of immune effectors, such as antimicrobial peptides (AMPs) -- host-encoded antibiotics that directly combat invading microorganisms [1]. For natural enemies, it is essential to overcome such host defences for success, thus setting the stage for antagonistic co-evolution. These evolutionary arms races have led to immune system genes typically evolving far more rapidly than other genes in the genome [2–5].

The genetically tractable model *Drosophila melanogaster* has been a workhorse of innate immunity, leading to the characterization of both the insect, and indeed animal, innate immune response [1]. *Drosophila* has also been of great importance to our understanding of the variability to which conserved genes may be expressed amongst closely related species, and how gene expression differences can result from interactions between genetics and environmental factors [6–8]. Upon the landmark sequencing of 12 Drosophila genomes in 2007, *Drosophila* researchers gained the ability to study the evolution of immune systems amongst closely related species [5, 9, 10]. An interesting pattern emerged, in that *Drosophila* immune signaling molecules were found to evolve rapidly, while immune effectors such as AMPs, did not [11, 12]. This pattern of AMP evolution was unexpected, given the importance of AMPs in the realized host response, and evidence for positive selection in AMPs of vertebrates [13–16] and social insects [17, 18].

**Fig. 1:**
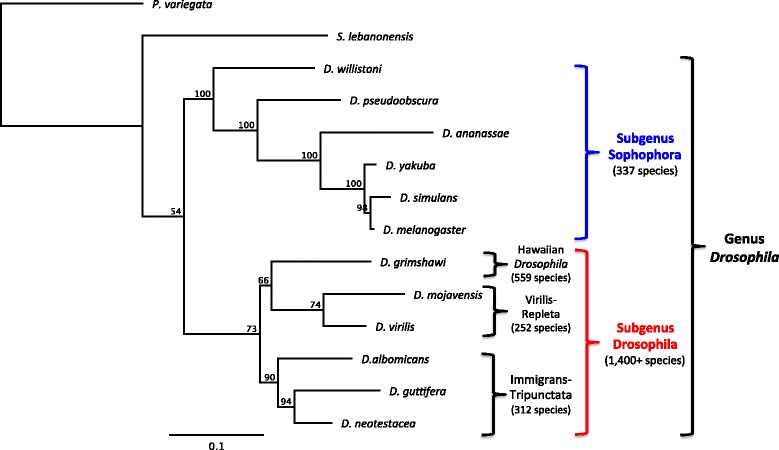
Phylogenetic relationships of the main lineages in *Drosophila*. Phylogeny constructed using maximum likelihood with concatenated Adh, amd, engrailed and glass protein sequences. Support values represent 100 bootstraps. Included species encompass much of the diversity of *Drosophila*, and form the basis of comparative work in this study. These sequences were generated from FlyBase curated protein translations and recently available sequence data from *D. albomicans, D. guttifera, D. neotestacea, S. lebanonensis,* and *P. variegata*. The subgenera Sophophora and Drosophila are estimated to have diverged 25-40 Ma (Obbard et al., 2012)

*Drosophila* is an incredibly speciose lineage, however, with a wide range of ecologies, life histories, and specialized natural enemies (Fig. 1). Yet there have been almost no functional studies on *Drosophila* immune genes in species other than *D. melanogaster* (but see [19–21]). Along with *D. melanogaster,* most of the original 12 sequenced genomes are found in the subgenus Sophophora [22], with only three species from the diverse and speciose subgenus Drosophila (*Drosophila grimshawi, D. virilis,* and *D. mojavensis*), and no work on immune evolution has been done in any of the 300+ members of the immigrans-tripunctata radiation in this subgenus. Sequence data has recently become available for three species of this radiation: the genomes of *Drosophila albomicans* and *D. guttifera* [21, 23], and the transcriptome of *Drosophila neotestacea* [24] providing the opportunity to investigate the immune capacities of this relatively unexplored lineage of the subgenus Drosophila.

We start to explore *Drosophila* immune diversity by characterizing the immune repertoire of *D. neotestacea*, a mushroom-breeding species in the immigrans-tripunctata radiation, whose interactions with its natural enemies, particularly parasitic nematodes, are well-studied [25]. *Drosophila neotestacea* has also recently garnered attention for harboring a *Spiroplasma* bacterial symbiont that protects against nematodes and parasitic wasps [26, 27]. In general, we found that genes involved in the immune response of *D. melanogaster* were highly conserved in *D. neotestacea*, but found surprising evolutionary patterns for AMPs. We investigated two of these AMPs in more detail, the *D. neotestacea* orthologues of diptericin and drosocin. Using phylogenetic analysis, we describe the evolutionary history of the *Drosophila* diptericin gene family and the conservation of the *Drosophila* AMP drosocin in subgenus Drosophila flies. We found that the diptericin gene family rapidly diverged in the ancestors of the genus *Drosophila*, leading to not two, but three distinct *Drosophila* diptericins. We confirmed that these AMPs are induced by bacterial challenge in *D. virilis*, but were surprised to find that diptericin in *D. neotestacea* was not induced by infection. Along with other recent studies [21, 28, 29], this work suggests that invertebrate AMPs are more dynamic than previously thought. Our results further highlight that conservation of immune genes, even in closely related species, does not necessarily imply conservation of the realized immune response.

## Methods

### The immune repertoire of *D. neotestacea*

Using a recently sequenced transcriptome, we characterized the immune repertoire of *D. neotestacea*. This transcriptome was generated in order to understand how the bacterial symbiont *Spiroplasma* protects *D. neotestacea* against parasitic nematode infection. In brief, symbiont-positive and negative flies were either infected or uninfected with nematodes, resulting in four treatments; eggs, larvae, pupae and adult flies were included [24]. This transcriptome is expected to include a broad range of immune-related genes, as it includes diverse infections including parasitic nematodes and trypanosomatid gut parasites; nematode-exposed flies would have also been exposed to microorganisms entering the haemolymph via punctures in the larval cuticle following nematode attack.

### Annotating the immune repertoire of *D. neotestacea*

We searched the *D. neotestacea* transcriptome using BLAST for immune genes that have been characterized in *D. melanogaster*. To generate a list of genes of interest, we conducted an extensive literature review to determine described constituents of the major *D. melanogaster* immune pathways: the Toll, Imd, JNK, JAK-STAT, and the melanization response pathways. We extracted *D. melanogaster* nucleotide sequences from FlyBase (vFB2015_04), and used BLASTn and tBLASTn to start to identify potential orthologous transcripts in the *D. neotestacea* transcriptome. When no significant hits (E < 0.1) were returned, we extracted corresponding orthologues from FlyBase for *D. virilis*, and/or *D. mojavensis,* and/or *D. grimshawi,* or from the *D. albomicans* [23]*,* or *D. guttifera* genome [30], and again used BLASTn and tBLASTn. Sources used to generate a list of immune genes were: Lagueux et al. [31], De Gregorio et al. [32], Lemaitre and Hoffman [1], Starz-Gaiano et al. [33], Valanne et al. [34], Zaidman-Remy et al. [35], Hughes [36], Marchal et al. [37], An et al. [38], Binggeli and Lemaitre [39], Amoyel et al. [40], Salazar-Jaramillo et al. [20], and Yamamoto-Hino et al. [41].

When a significant BLAST hit was returned, the nucleotide sequence of the *D. neotestacea* transcript was then aligned with sequenced *Drosophila* orthologues as annotated by FlyBase for initial exploration. Transcript(s) were also codon-aligned with orthologues from *D. melanogaster* and *D. virilis* to confirm amino acid sequence similarity. In instances where amino acid sequence poorly resembled both *D. melanogaster* and *D. virilis* orthologues, or when multiple transcripts closely resembled these sequenced orthologues, additional genes from diverse *Drosophila* were extracted from FlyBase to provide outgroups for comparison. These genes were aligned using MUSCLE [42], followed by phylogenetic analysis using the Neighbour-joining method (1000 bootstraps); all analyses were performed in Geneious 7. When a *D. neotestacea* putative orthologue clustered with those from subgenus Drosophila flies, we considered this gene to be the true orthologue of the corresponding *D. melanogaster* gene.

### Checking for presence of missing genes in the *D. neotestacea* genome

*Drosophila melanogaster* immune genes may be absent from the *D. neotestacea* transcriptome for a number of reasons; for instance, immune genes may be restricted to *D. melanogaster* and relatives (e.g. subgenus Sophophora, melanogaster subgroup). Alternatively, immune genes may be absent in the transcriptome despite their conservation in relatives of *D. neotestacea*. In this instance, there are two possibilities for this lack of expression. First, these immune genes may be absent from the *D. neotestacea* genome. Second, these immune genes might be present in the *D. neotestacea* genome, but were not expressed in the transcriptome.

Four genes were absent from the *D. neotestacea* transcriptome that were expected to be present. We followed up on these absent genes by designing PCR primers using sequenced *Drosophila* genomes, and tested these primers on a diversity of *Drosophila* including: *D. neotestacea, D. falleni,* and *D. subobscura*. Once the *D. guttifera* genome became available, we instead used the *D. albomicans* and *D. guttifera* genomes to determine if these genes were present or absent in the ancestor of *D. neotestacea*.

Three of the four genes apparently absent from the *D. neotestacea* transcriptome were short AMP genes. Due to the length of these AMP genes, BLAST was often unable to recover orthologues when searching the *D. albomicans* and/or *D. guttifera* genomes. To overcome this challenge, the synteny of the gene of interest in *D. melanogaster* was determined, and we then used longer genes that flanked the gene of interest as queries for BLAST searches. If an orthologue of a gene flanking the gene of interest was found in either the *D. albomicans* or *D. guttifera* genome, a manual search for the gene of interest was then conducted by identifying potential ORFs or conserved domains in the appropriate upstream or downstream gene region.

### PCR primers, protocols, cloning, and sequencing

Primers used to successfully amplify immune genes absent from the *D. neotestacea* transcriptome can be found in Additional file 1: Table S1. Polymerase chain reactions were 12.5 μL in volume (1.25 μL 10× PCR mastermix, .2 mM dNTPs, 1.5 mM MgCl2, 0.625 μL of 0.25 μM forward and reverse primers, and 0.31 units of taq polymerase (Applied Biological Materials) with 0.5 μL of DNA template). All PCR products were Sanger sequenced to confirm that we were amplifying the correct sequence, and in the case of *D. neotestacea* genes of interest, to confirm transcriptome sequence. Sanger sequencing of PCR products was carried out by Macrogen USA. Sequences have been deposited in GenBank, under the following accession numbers: KX469340-KX469349.

### Searching for novel immune genes in *D. neotestacea*

As the overwhelming majority of immune study in *Drosophila* has been done using *D. melanogaster*, it is possible that *D. neotestacea* transcribes as-yet uncharacterized immune genes that are restricted to *D. neotestacea* and related lineages (e.g. subgenus Drosophila). To examine this possibility, we looked for transcripts with homology to manually curated immune gene families from ImmunoDB [43]. Immune gene families were aligned with MUSCLE or MAFFT [44], after which profile HMMs were generated using hmmbuild in HMMer 3.1 (http://hmmer.org). We also included an alignment of Nimrod-like proteins extracted from a BLAST search using *D. melanogaster* nimrod on GenBank to generate a Nimrod-like domain profile, which is absent from ImmunoDB. We then searched all potential ORFs from the *D. neotestacea* transcriptome against these 39 profile HMMs using hmmsearch. Resulting significant matches were filtered for those that did not have an identified *Drosophila* orthologue from annotation by Hamilton et al. [24]. Finally, as the *D. neotestacea* transcriptome contains transcripts from *Drosophila*, nematodes, and trypanosomatids, we filtered these remaining ORFs for likely *Drosophila* transcripts, as annotated by Hamilton et al. [24]. The resulting list therefore contained likely *Drosophila* genes that lacked an orthologue in annotated *Drosophila* genomes on FlyBase.

### Phylogenetic analysis of diptericin genes

We extracted annotated diptericins from FlyBase, and used BLAST to search GenBank, and recently sequenced drosophilid and dipteran genomes [45] for diptericin genes from a diversity of flies. The well-conserved glycine-rich domains (G domains) of these diptericins were then codon-aligned using MUSCLE. We used PhyML to construct a maximum likelihood phylogeny for these diptericin sequences with an AIC-selected best model of nucleotide substitution determined by Datamonkey.org model selection [46]. Diptericins from *Mayetiola destructor*, *D. ananassae* (Dana\GF11125) and *D. simulans* (Dsim\GD11418) were excluded from this phylogeny due to very long branches.

### Synteny of diptericins in sequenced *Drosophila* genomes

We found three clades of *Drosophila* diptericins (hereon referred to as either Diptericin (Dpt) A, B, or C). To determine evolutionary relationships of *Drosophila* diptericins, we inspected the diptericin gene regions of drosophilid flies using FlyBase and sequenced drosophilid genomes. We extracted the diptericin-containing scaffold and manually searched for conserved diptericin motifs in this gene region to identify diptericin duplications if present. In its current genomic scaffold assembly, the signal peptide and P domain of the *D. guttifera* DptC gene was unavailable, and thus the N-terminus of this diptericin is not included in this analysis. Also, the intergenic region between the two *D. guttifera* diptericins was not fully sequenced, and thus the reported length for this intergenic region represents currently available sequence.

We aligned diptericin gene regions of *D. melanogaster* and *D. virilis* to related flies to generate an alignment encompassing divergent diptericins in diverse drosophilids. For some species, additional diptericin duplications were present, and we used flanking genes to determine the ancestral gene copy for alignment purposes. The *P. variegata* genome encodes two DptB orthologues not found on the same genomic scaffolds.

### Positive selection on *Drosophila* diptericins

Intrigued by the degree of amino acid sequence similarity amongst *Drosophila* diptericins, we investigated rates of synonymous and non-synonymous change (dN/dS) in the diptericin G domain. We used Branch-site REL (BSR) [47] implemented in Datamonkey.org to identify lineages with elevated dN/dS in the diptericin G domain. To rule out the possibility that our results were sensitive to the presence of certain divergent diptericins, we repeated the analysis while removing divergent sequences.

### Characterizing drosocin in the subgenus Drosophila

We recovered a drosocin-like gene (hereafter referred to as “drosocin”) with multiple tandem drosocin-domain repeats in the *D. neotestacea* transcriptome. We extracted similar drosocin gene sequences from sequenced genomes combining BLAST and manual gene region curation. Many, but not all, drosocin ORFs contained multiple tandem repeats, and so we aligned unique repeats of this drosocin gene with drosocins found in subgenus Sophophora flies using MUSCLE.

### Fly cultures used in infection experiments

For infection experiments, we used a strain of *D. neotestacea* originally collected in W. Hartford, Connecticut, in 2006. The *D. virilis* strain used in this study was donated by Brent Sinclair (Western University, Canada), and the *D. melanogaster* strain (Oregon-R) used in this study was donated by Bruno Lemaitre (EPFL, Switzerland). All strains used were *Wolbachia* and *Spiroplasma* negative. All species were maintained at 21 °C with a 12-h light:dark cycle on Instant Drosophila Medium (Carolina Biological Supply). Approximately 10 females were allowed to lay on 1/2 tsp. Instant Drosophila Medium (1 tsp. for *D. virilis*) with 1:1 water; *D. neotestacea* vials were supplemented with ~0.5 g *Agaricus bisporus*. Newly emerged males were then collected daily and kept in isolation from females for 3–4 days on ~1/2 tsp Instant Drosophila medium with 1:1 water. All adults used in infection assays were 3–4 day old virgin males.

### Immune challenge with Gram-negative bacteria (IMD pathway challenge)

Gram-negative bacteria induce the Imd immune pathway in *Drosophila* [1]. For our Imd pathway challenge, we used a pathogenic *Serratia* strain closely related to the soil bacterium *Serratia marcescens*, and isolated from mycophagous *Drosophila* cultures (Additional file 2: Figure S4). Bacteria were grown overnight at 37 °C and diluted in Luria-Bertani broth prior to wounding experiments.

Flies were lightly anaesthetized on CO_2_ and wounded in the left side of the thorax above the wing with a 0.6 μm tip tungsten needle. For septic woundings, this needle was dipped in OD_600_ = 0.15 ± 0.05 *Serratia* in Luria-Bertani broth. Flies were then left to recover in a clean polystyrene vial for 30 min prior to transfer to a vial containing ~1/2 tsp Instant Drosophila medium with 1:1 water. Six hours post-wounding, flies were flash frozen in liquid nitrogen and kept at −80 °C until RNA was extracted.

This experiment was performed three times for *D. neotestacea* and *D. virilis*, and once for *D. melanogaster*. Additionally, for one replicate experiment using *D. virilis* and *D. neotestacea* we also examined flies that were anaesthetized on CO_2_ and not wounded to provide a reference treatment for differences in AMP expression incurred by sterile wounding alone.

### Immune challenge with fungi (Toll pathway challenge)

Pathogenic fungi induce the Toll signaling pathway in *Drosophila* [1]. We used the entomopathogenic fungus *Beauveria bassiana* (strain UAMH 1514) for our Toll pathway challenges. *Beauveria* cultures were provided by Will Hintz and Jon Leblanc (University of Victoria, Canada), and were grown on Potato-dextrose agar at 27 °C for one week until fungus was sporulating prior to exposures.

Flies were lightly anaesthetized on CO_2_ and transferred to either a sterile Potato-dextrose agar petri dish, or one containing sporulating *Beauveria* culture. Dishes were then shaken by hand for 30 s to cover the flies in fungal spores; we confirmed flies had been exposed to fungal spores using a dissecting microscope shortly after shaking. Flies were left to recover in a clean polystyrene vial for 30 min prior to transfer to a vial containing ~1/2 tsp Instant Drosophila medium with 1:1 water. Twenty-four hours post-exposure, flies were flash frozen in liquid nitrogen and kept at −80 °C until RNA was extracted.

This experiment was performed three times for *D. virilis*, and twice for *D. melanogaster* and *D. neotestacea*.

### RNA extraction and cDNA synthesis

We extracted RNA from six to eight flies per treatment using Trizol-LS (Invitrogen) with the manufacturer’s protocol. Individual flies were added to microfuge tubes containing 300 μL Trizol and 5–15 0.1 mm silica/zirconia beads, and bead-beat for 3 s (BioSpec MiniBeadbeater 16). Following extraction, pellets were re-suspended in 20 μL RNAse-free water for five minutes at room temperature.

RNA purity was measured using 1 μL RNA on a Nanodrop 2000 Spectrophotometer (Thermo Scientific). The remaining 19 μL from each sample were then DNAse treated (Thermo Scientific DNAse I) according to the manufacturer’s protocol, with the DNAse heat inactivated. Extraction quality was assessed by agarose gel electrophoresis.

DNAse-treated RNA was reverse-transcribed using Applied Biological Materials 5X All-In-One RT MasterMix. Reverse transcription reactions were 20 μL containing 4 μL RT MasterMix and 16 μL of RNA in RNAse-free H_2_O with 300–1000 ng total RNA.

### qPCR for gene expression and data analysis

Levels of expression for genes of interest were quantified using the qPCR primers listed in Additional file 1: Table S1. Primers were designed using Primer3, and primer efficiency was verified using a 5 × 5 fold dilution series; primer efficiencies are reported in Additional file 1: Table S1. All qPCR reactions used the following thermal cycling conditions: 95 °C for 10 min, then 35 cycles of 95 °C for 15 s followed by 60 °C for 45 s, with the product verified by melt curve analysis, as well as Sanger sequencing (Macrogen USA) once for each primer set. We used Applied Biological Materials, EvaGreen 2X qPCR MasterMix according to manufacturers protocol, with a BioRad CFX96 qPCR thermal cycler.

For all immune challenges, we assayed the expression of each fly’s respective diptericin orthologue. In *Serratia* challenges, we also assayed attacin B (AttB) and drosocin. In *Beauveria* challenges, we also assayed a bomanin (Bom) gene (CG5791 in *D. melanogaster* and its respective orthologues in *D. virilis* and *D. neotestacea*), drosomycin in *D. melanogaster*, and drosocin in *D. neotestacea* and *D. virilis*.

For all qPCR reactions, target genes were run alongside a normalizing control gene (RpL28, RpL32, and RpL11 for *D. neotestacea*, *D. melanogaster*, and *D. virilis* respectively). Each reaction was run in triplicate, and replicates were considered consistent if the threshold cycle (C_T_) of each replicate was contained within a 0.5 C_T_ boundary.

Gene expression analysis was performed using the 2^ΔΔCT^ method [48], and we report these data as boxplots using the ΔC_T_ values (ΔC_T_ = C_T_ target gene – C_T_ reference gene). Two-sample Welch’s T-tests of ΔC_T_ values were used to determine differences in expression profile in R 3.1 statistical software.

## Results

### The immune repertoire of *D. neotestacea*

**Fig. 2:**
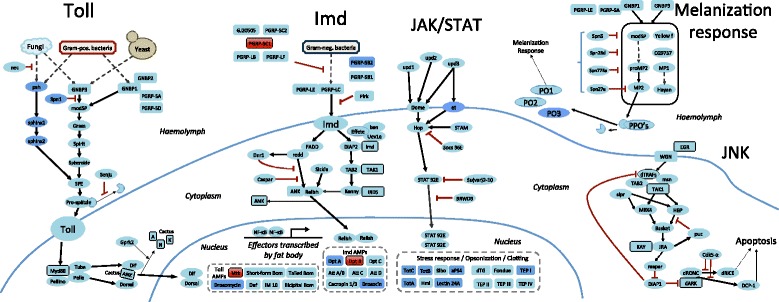
The immune repertoire of *D. neotestacea*. This diagram is colour-coded to indicate: i) genes predicted to be present, and that were recovered in the transcriptome (*light blue*), ii) genes predicted to be absent in the transcriptome (because they are restricted to the subgenus Sophophora), and that were absent (*dark blue*), and iii) genes predicted to be present in the transcriptome, but were absent (*red*). The vast majority of immune genes matched predicted patterns of conservation in *D. neotestacea*, with the exception of the effector genes DptB and Mtk, and the Imd pathway inhibitor PGRP-SC1

We found that 105 out of 108 genes expected to be involved in *Drosophila* immune pathways were present in the *D. neotestacea* transcriptome (Fig. 2). We did not recover the AMP metchnikowin (Mtk), nor could we amplify it from genomic DNA. We found Mtk in the genomes of the subgenus Drosophila flies *D. mojavensis, D. virilis,* and *D. albomicans*, but not *D. guttifera*; we were unable to determine if it is truly absent in *D. guttifera*, or if this absence is instead an artefact of the current genomic assembly. We did not recover an orthologue of PGRP-SC1 in the *D. neotestacea* transcriptome, but found that *D. neotestacea* harbours two copies of PGRP-SC2; PGRP-SC1 and SC2 have been shown to have mutually exclusive activities in *D. melanogaster* (Additional file 3: Figure S1) [49, 50]. Finally, diptericin B was not found in the *D. neotestacea* transcriptome, but we subsequently found an interesting pattern of diptericin evolution (see below).

### Drosocin in the subgenus Drosophila

**Fig. 3:**
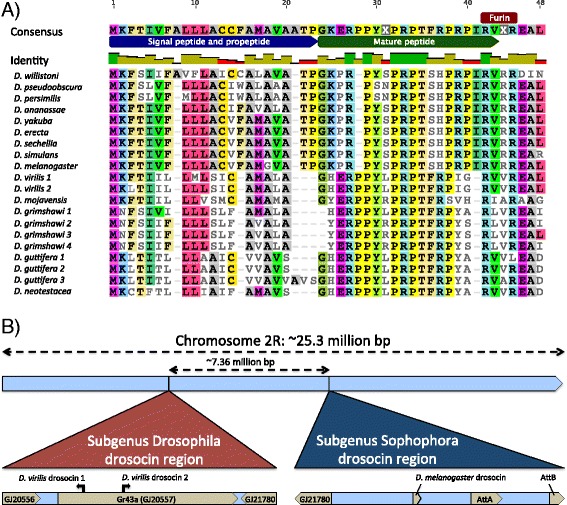
Drosocin in the subgenus Drosophila. **a** The subgenus Drosophila drosocin has a strongly conserved ERPPY motif at the proline-rich N-terminus, followed by the drosocin domain PRPT, which includes a critical threonine residue. This domain is followed by furin-like cleavage sites (annotated as “Furin”). The presence of both furin-like cleavage sites and the key threonine residue indicate that this transcript is likely processed to produce multiple copies of a mature drosocin peptide glycosylated at its PRPT threonine. This alignment presents the signal peptide and first drosocin repeat in each species, and does not include tandem drosocin repeats, which vary in number and sequence depending on species. **b** Drosocin in the subgenus Drosophila is found within the gene region of DSCAM1 and Gr43a, ~7.36 million base pairs displaced from the drosocin gene region in the subgenus Sophophora. The *D. virilis* Gr43A gene region is included here

We searched for potentially novel immune genes in *D. neotestacea* with HMMer 3.1 using immune gene profiles. This search method recovered one immune gene of interest in the *D. neotestacea* transcriptome: a *D. neotestacea* drosocin; drosocin was previously thought to be absent in the subgenus Drosophila [19]. We further found orthologues of this *D. neotestacea* drosocin in other subgenus Drosophila flies. The signal peptide of this drosocin gene almost-perfectly matches that of drosocin genes in Sophophora species, however the *D. neotestacea* transcript contains multiple tandem repeats of drosocin protein domains (Fig. 3); this pattern of drosocin domain tandem repeats was recovered in some, but not all, of the other sequenced subgenus Drosophila genomes. Interspersed between each drosocin domain repeat are furin-like cleavage sites (e.g. RVVR), suggesting that the translated protein is likely cleaved into multiple mature drosocin peptides (Fig. 3a). Finally, while drosocin is found just upstream of the attacin gene region on chromosome 2R in *D. melanogaster*, subgenus Drosophila drosocin occurs in the gene region of the *Drosophila* down syndrome cell adhesion molecule (DSCAM1) and gustatory receptor 43a (Gr43a), ~7.36 million base pairs displaced from the attacin gene region, but still on chromosome 2R (Fig. 3b). We later confirmed that this subgenus Drosophila drosocin responds to immune challenge (see below).

### Diptericin in *D. neotestacea* and other Diptera

We did not recover a diptericin B orthologue in the *D. neotestacea* transcriptome, but found DptB sequences in both the *D. albomicans* and *D. guttifera* genomes; however the DptB molecule in *D. guttifera* has been pseudogenized by mid-exon frame shifts in both the diptericin P domain and G domain. We did however find a divergent diptericin previously annotated as diptericin B by Hamilton et al. [24] that, upon further inspection, was not highly similar to either of the diptericin genes (DptA and DptB) in *D. melanogaster*.

**Fig. 4:**
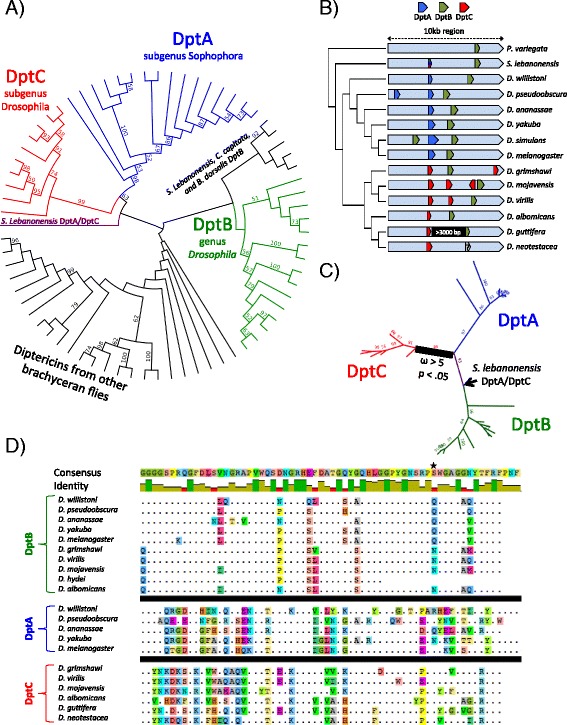
The subgenus Drosophila encodes a highly divergent diptericin. **a** Maximum likelihood tree generated using a codon alignment of the well-conserved diptericin G domain from assembled diptericins of diverse brachyceran flies. Support values represent consensus from 100 bootstraps. Four distinct diptericin clades emerge, including three in the genus *Drosophila*: DptA (subgenus Sophophora), DptB (genus *Drosophila*), and a diptericin restricted to the subgenus Drosophila that we term DptC. **b** Synteny of diptericins in the genomes of sequenced drosophilids. DptA and DptC both occur upstream of DptB. We include *D. neotestacea* despite lacking a sequenced genome to indicate the lack of DptB recovered from the transcriptome. Diptericin B in *D. guttifera* is pseudogenized, and the intergenic region between DptC and DptB is listed as “>3000 bp” due to its current assembly. In *S. lebanonensis*, the DptA/DptC syntenic orthologue is present, but does not bear great similarity to any diptericin clade. **c** Summary phylogeny of Branch-site REL (BSR) analyses using only drosophilid diptericin G domains. Likelihood-ratio tests for branches with dN/dS > 1 consistently identified the branch leading to DptC as having evolved under diversifying (positive) selection (*p* < .05). Additional file 4: Figure S2 provides an example BSR analysis. **d** Amino acid alignment of the diptericin G domain from *Drosophila* diptericins. Numerous fixed differences are unique to each clade, particularly in the Gly22-Asp45 region. Greater conservation is observed in the Asn46-on region. The polymorphism at residue 71 described by Unckless et al. [21, 29] is indicated by a ★, and displays conserved differences amongst diptericin lineages in sequenced genomes

To determine the identity of this divergent *D. neotestacea* diptericin, we inspected diptericins from diverse Diptera, and using phylogenetic analysis, found that the *D. neotestacea* diptericin belongs to a clade of diptericins restricted to the subgenus Drosophila we term diptericin C (DptC) for clarity of discussion (Fig. 4a). Intriguingly, DptC genes clustered on a long branch separate from other *Drosophila* diptericins; to determine their evolutionary history we investigated the genomic positions of DptC genes in sequenced subgenus Drosophila flies.

We found that DptC genes are encoded as only one exon, and are syntenic with the one-exon DptA genes of subgenus Sophophora flies, upstream of the two-exon DptB in the diptericin gene region (Fig. 4b). Moreover, we recovered this one-exon diptericin in the outgroup drosophilid *Scaptodrosophila lebanonensis* (Drosophilinae, Drosophilidae), but not in *Phortica variegata* (Steganinae, Drosophilidae), or in *Ephydra gracilis* (Ephydridae) (Fig. 4b). We recovered intact DptB genes in all sequenced drosophilids barring *D. neotestacea* (absent from transcriptome) and *D. guttifera* (pseudogenized).

The extreme divergence of these syntenic orthologues prompted us to search for signatures of positive selection in *Drosophila* diptericins (i.e. DptA, DptB, and DptC). Using Branch-site REL, we found that the branch leading to the DptC clade diverged under diversifying selection (likelihood ratio test (LRT); *p* < 0.05) (Fig. 4c). This result was robust to removal of the more divergent diptericins from the analysis. Additionally, we recovered some support for the hypothesis that DptA also diverged from DptB (LRT = 12.25; *p* = .017) through diversifying selection in the ancestor of the subgenus Sophophora (Additional file 4: Figure S2).

Comparing DptA, DptB, and DptC protein sequences, we found that the diptericin G domain has undergone considerable modification unique to but conserved within each diptericin clade (Fig. 4d). The Gly22-Asp45 region of the diptericin G domain was previously hypothesized by Cudic et al. [51] to be the region responsible for diptericin’s antibacterial activity. We found that 15 of the codons in this 23-residue region show lineage-restricted conserved differences, while the Asn46 to C-terminal region of the G domain shows greater conservation amongst *Drosophila* diptericin clades. Interestingly, in *D. melanogaster* and *D. simulans*, Unckless et al. [21] found that balancing selection is maintaining a polymorphism at residue 69 of the diptericin G domain (corresponding to residue 71 from Cudic et al. [51]), and that whether serine or an arginine was found at this site strongly affected resistance to pathogenic bacteria. We found this residue to be different between, but conserved within, lineages of DptB and DptC, yet polymorphic in DptA (Fig. 4d). We also found that diptericins in certain *Drosophila* species lacked a positively charged G domain (Additional file 5: Table S2); antimicrobial peptides are thought to require a positive net charge for bacterial killing [1].

### AMP gene expression in subgenus Drosophila flies

We sought to determine if DptC and drosocin in subgenus Drosophila flies responded to immune challenge by Gram-negative bacteria and fungi. We found that drosocin was induced by Gram-negative bacterial challenge in both *D. virilis* and *D. neotestacea* (see below). We also found that while DptC was induced by Gram-negative challenge in *D. virilis*, surprisingly, DptC was not at all induced in *D. neotestacea* (see below). These two AMPs were not strongly induced by fungal challenge (see below).

### Immune challenge with Gram-negative bacteria (Imd pathway challenge)

**Fig. 5:**
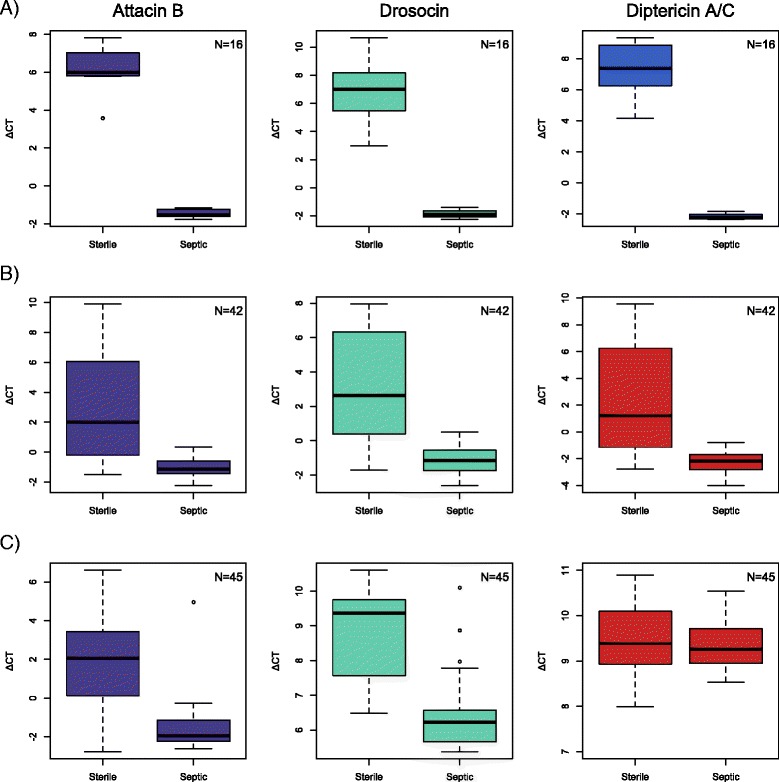
AMP gene expression following *Serratia* challenge. AMP expression was measured six hours after either sterile wounding (Sterile) or *Serratia* bacterial challenge (Septic) in **a** *D. melanogaster*, **b** *D. virilis*, and **c** *D. neotestacea*. N represents total sample size. Attacin B and drosocin were strongly induced in all three species (*p* < 0.001). However while DptA and DptC were strongly induced in *D. melanogaster* (*p* < 0.0001), and *D. virilis* (*p* < 0.0001) respectively, DptC in *D. neotestacea* was not (*p* = 0.42)

As expected, the Imd pathway-regulated genes AttB, drosocin, and DptA were induced by *Serratia* challenge in *D. melanogaster* (*t*(7.46) = 16.65, *p* < 0.0001, *t*(7.23) = 10.53, *p* < 0.0001, *t*(7.17) = 15.11, *p* < 0.0001, respectively) (Fig. 5a). This pattern of induction was also found in *D. virilis* for AttB, drosocin, and DptC (*t*(20.15) = 4.74, *p* < 0.0005, *t*(21.45) = 5.67, *p* < 0.0001, *t*(20.31) = 5.07, *p* < 0.0001, respectively) (Fig. 5b). However in *D. neotestacea*, while AttB and drosocin were both induced by *Serratia* infection (*t*(34.64) = 5.37, *p* < 0.0001; *t*(36.13) = 5.71, *p* < 0.0001, respectively), DptC (*t*(28.79) = 0.82, *p* = 0.42) was not (Fig. 5c). We also found that while AttB experiences an increase in expression in both *D. virilis* and *D. neotestacea* upon sterile wounding, we did not observe this level of induction in either *D. virilis* or *D. neotestacea* for drosocin and DptC (Additional file 6: Figure S3).

### Immune challenge with fungi (Toll pathway challenge)

**Fig. 6:**
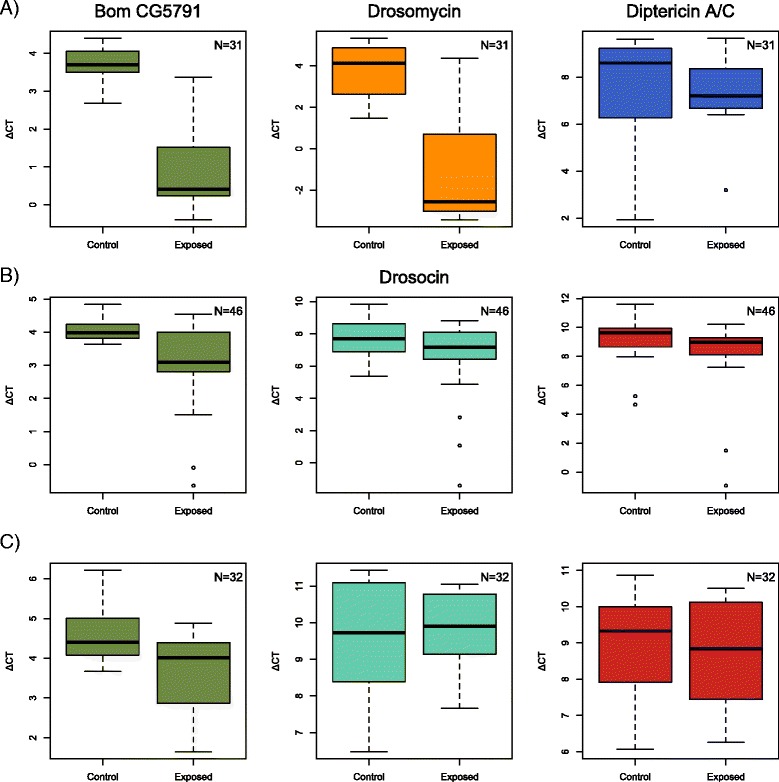
AMP gene expression following *Beauveria* challenge. AMP expression was measured 24 h after either fungus-free controls or *Beauveria* challenge in **a** *D. melanogaster*, **b** *D. virilis*, and **c** *D. neotestacea*. N represents total sample size. Both Bom CG5791 and drosomycin were strongly induced in *D. melanogaster* (*p* < 0.0001). The orthologues of Bom CG5791 were induced in both *D. virilis* and *D. neotestacea* (*p* < .005), though to a lesser extent than in *D. melanogaster*. Drosocin and DptC in *D. virilis* and *D. neotestacea* were not strongly induced by *Beauveria* challenge; drosocin differential expression in *D. virilis* was marginally significant (*t*(28.11) = 2.18, *p* = .038). Diptericin A in *D. melanogaster* was not induced by *Beauveria* challenge (*p* = 0.99)

Due to the absence of many canonical Toll-regulated genes in the *D. neotestacea* transcriptome, we used a member of the recently described bomanin (Bom) gene family [52] to serve as a read-out of Toll pathway expression, the *D. melanogaster* Bom CG5791; Bom CG5791 was induced by septic injury and fungal infection in De Gregorio et al. [32]. In *D. melanogaster*, *Beauveria* infection strongly induced the Toll-regulated AMP drosomycin and also Bom CG5791 (*t*(19.05) = 5.59, *p* < .0001, *t*(17.85) = 8.45, *p* < .0001, respectively), but DptA expression was unaffected (*t*(24.86) = –0.01, *p* = 0.99) (Fig. 6a). This pattern of induction confirmed that CG5791 behaved as would be expected of a Toll-regulated AMP. We used this bomanin in *D. virilis* (GJ23146) and *D. neotestacea* (TSA Accession: GDUH01009588) as a read-out to confirm expression of the Toll pathway in our *Beauveria* infections. We found that *Beauveria* infection induced CG5791 in both *D. virilis* and *D. neotestacea* (*t*(23.18) = 3.55, *p* < .005, (*t*(27.42) = 3.24, *p* < .005, respectively) (Fig. 6c), though the change in expression (~1.1 ΔC_T_) was not as large as in *D. melanogaster* (2.7 ΔC_T_). Neither DptC nor drosocin were induced in *D. neotestacea* upon fungal exposure (*t*(29.94) = 0.50, *p* = 0.62, (*t*(26.96) = –0.42, *p* = 0.68, respectively) (Fig. 6). DptC was not induced in *D. virilis* (*t*(32.09) = 1.41, *p* = 0.17), though drosocin appeared to be induced in a few individuals (Fig. 6b); two *D. virilis* individuals had elevated bomanin, DptC, and drosocin, expression in the “Exposed” treatment*.*

## Discussion

Using a recently sequenced transcriptome as a starting point, we characterized the immune repertoire of *D. neotestacea*, a mushroom-breeding species in the subgenus Drosophila, which is estimated to have diverged from *D. melanogaster* and the subgenus Sophophora approximately 25–40 Ma ago [53]. The vast majority of immune genes were conserved and expressed in this transcriptome, with some interesting exceptions, particularly among AMPs. This finding opens a window into the diversity of the realized *Drosophila* immune response. The diversity of AMPs conserved in the subgenus Drosophila was unexpected and parallels renewed interest in *Drosophila* and arthropod AMPs [12, 21, 29]. Previous explorations of *Drosophila* immune evolution did not recover signals of selection in AMPs, but rather signaling pathway intermediates [4, 5, 9, 11]. As such, the predominant view of insect immune evolution holds that insect AMPs do not evolve rapidly, in contrast with many studies documenting balancing selection on AMPs in vertebrates (e.g. [13–16]). This view of AMP evolution in *Drosophila* may have resulted from two factors in particular. First, AMPs are often exceedingly short and therefore challenging to study using standard methods to examine homology, divergence, and signals of natural selection. Second, AMPs have been characterized in relatively few arthropod lineages.

The divergent evolution of diptericin, including finding a lineage (DptC) that is as deeply branching and diverse in *Drosophila* as DptA and DptB, is surprising. Diptericin has been the canonical readout for the Imd pathway in flies (induced by Gram-negative bacteria), and diptericins are especially well characterized in *D. melanogaster* [54, 55], although their structure and mode of action are yet to be fully described [1]. It would be very interesting to determine what are the functional consequences of diptericin variation, and whether the numerous conserved differences distinct to each diptericin lineage underlie unappreciated diversity in immune capacities of these genes, possibly representing adaptation to ecologically relevant natural enemies. Indeed, an interesting recent study found that variation at a single residue in DptA had striking consequences on the ability of *D. simulans* and *D. melanogaster* to resist infection by *Providencia* bacteria [21]. We found this residue to be highly variable across *Drosophila* (Fig. 4d).

Although DptC behaved as expected in response to microbial challenge in *D. virilis,* its lack of induction upon *Serratia* challenge in *D. neotestacea* is surprising and warrants further study. Traditionally, conservation of immune genes has been interpreted as representing a conservation of immune function. Yet *D. neotestacea* employs neither DptC, nor as far as we can tell, any other diptericin in its response to *Serratia* challenge. However DptC in *D. neotestacea* can be induced, as two adult females had elevated levels of DptC in Hamilton et al. [24], and they suspected these elevated DptC levels to have resulted from a cryptic bacterial infection, although we note that we only challenged adult males. Alternatively, tissue-specific AMP expression could account for the lack of DptC induction in *D. neotestacea* [56]. If DptC were involved in the local immune responses of surface epithelia such as in tracheae or the gut, septic wounding of the thorax may not induce DptC. Regardless, the lack of diptericin employed in response to *Serratia* infection implies that the *D. neotestacea* AMP arsenal combats certain bacteria without using any diptericins. As attacins and diptericins have common ancestry [54], it may be useful to consider the potentially redundant roles these AMPs play in the *Drosophila* immune response; attacin was highly expressed following *D. neotestacea* exposure to *Serratia*. It would also be interesting to challenge *D. neotestacea* with other gram-negative bacteria to see if DptC fails to be induced in general.

We also recovered and provide the first description, to our knowledge, of the AMP drosocin in the subgenus Drosophila. We found that in many species in this subgenus, drosocin contains multiple tandem repeats of the domains ERPPY and PRPT, which are likely proteolytically cleaved to produce multiple drosocin molecules at furin-like cleavage sites (e.g. RVVR) found between each repeat (Fig. 3). There are well-documented trade-offs with respect to mounting a host defence to infectious microbes [57–59], leading to the hypothesis that AMP expression should be optimized to expend only the minimum amount of energy required for an effective host defence [52]. The tandem-repeat drosocin genes of closely related subgenus Drosophila flies may allow researchers to test this hypothesis if flies optimize levels of drosocin expression and mature peptides produced. Additionally, there are many sequence differences amongst drosocins in the subgenus (Fig. 3), which may imply balancing selection [29]; we did not perform selection analyses for drosocin as the tandem-repeat structure of subgenus Drosophila drosocins make alignments somewhat subjective.

Our comparative approach allowed us to better characterize the conservation of metchnikowin (Mtk), a canonical read-out of the Toll pathway in *D. melanogaster*. Metchnikowin orthologues are annotated in FlyBase (vFB2015_04) in most species in the subgenus Sophophora (except obscura group species) as well as in *D. grimshawi*. Using manual curation, followed by BLAST, we recovered Mtk in the obscura group species *D. pseudoobscura, D. persimilis*, and *Drosophila miranda*, the subgenus Drosophila flies *D. virilis, D. mojavensis,* and *D. albomicans*, as well as *D. busckii*, *S. lebanonensis,* and *P. variegata*. We were not able to recover Mtk from *D. neotestacea* and D. *guttifera*. However, given our recovery of diptericin, drosocin, and Mtk from subgenus Drosophila flies, it seems that conservation of *D. melanogaster* AMPs is more widespread than previously described ([19]; FlyBase vFB2015_04).

## Conclusions

This study lends further support to the idea that invertebrate AMPs evolve rapidly, and that *Drosophila* species harbor a diverse repertoire of AMPs with potentially important functional consequences. As such, investigating AMP polymorphisms promises to be an exciting field of research in coming years, both to understand factors contributing to susceptibility to infection [29], and perhaps even to provide templates for the discovery and development of novel antibiotics [60].

## Additional files

Additional file 1: Table S1. PCR primers used in expression analyses and amplification of immune gene DNA from diverse *Drosophila*. (XLSX 39 kb)
Additional file 2: Figure S4. Our *Serratia* strain is an oral pathogen of *D. neotestacea* related to *Serratia marcescens*. A) Flies were fed on mushroom agar with either 100uL of Luria-Bertani Broth (Control) or OD600 = 0.1 *Serratia* solution (Exposed) for 6 h, prior to transfer to sterile mushroom agar vials. Flies were turned over into new agar vials every 4 days, and mortality was recorded daily. Crosses indicate flies that were lost unrelated to treatment. Flies exposed to *Serratia* suffered significantly shorter lifespans compared to control treatments (*n* = 212; LR test: *χ* ^2^ = 11.8, *p* = 5.90e–4; GW test: *χ* ^2^ = 13.3, *p* = 2.66e–4). B) Maximum likelihood tree (100 bootstraps) of the isolated *Serratia sp.* 16S gene highlighted in red, with *Rahnella sp.* included as an outgroup. (PDF 390 kb)
Additional file 3: Figure S1. Phylogenetic analysis of PGRP-SC1 and SC2 amino acid sequences using maximum likelihood. Support values indicate 100 bootstraps. *Drosophila neotestacea* has two PGRP-SC genes. The *D. albomicans* PGRP-SC1 signal peptide sequence is unresolved, and the current scaffold assembly in the *D. guttifera* genome does not contain the anterior region of its PGRP-SC1 orthologue. (PDF 45 kb)
Additional file 4: Figure S2. Example branch-site REL analysis of *Drosophila* diptericins. Branch colour indicates the strength of selection, with red corresponding to dN/dS > 5, grey to dN/dS = 1, and blue to dN/dS = 0. The width of the colour on each branch represents the proportion of sites in the corresponding class. Bolded branches indicate branches that evolved under positive selection. In this BSR analysis, there is strong evidence that the root branch of the DptC clade diverged through positive selection (*p* < 0.001); there is also some evidence for positive selection at the divergence of DptA from DptB (*p* = .017). The codon alignment used in this analysis does not include *D. ananassae* diptericins, as *D. ananassae* DptA clustered with *D. willistoni* DptA on extremely long branches. This *D. ananassae* diptericin amino acid sequence can be seen in Fig. 4d, and consequences on net charge of *D. ananassae* DptA are shown in Additional file 5: Table S2. (PDF 47 kb)
Additional file 5: Table S2. Net charges of DptA or DptC domains from *Drosophila* diptericins. Net charges were calculated using Protein Calculator v3.4 (http://protcalc.sourceforge.net/), and are given for each domain. DptC shows extensive differences at the amino acid sequence level. These differences have resulted in more extreme charges on each domain in the DptC molecule. DptC molecules generally have more negative P domains (except for *D. albomicans*), and more positive G domains, resulting in relatively similar net charges to DptA (except for *D. albomicans*). (XLSX 35 kb)
Additional file 6: Figure S3. AMP gene expression following no-wound controls in (A) *D. virilis* and (B) *D. neotestacea*. Treatments involved a sterile wound (Sterile), *Serratia* bacterial challenge (Septic), or a no-wound control (No Wound). Comparing sterile wound treatments to no-wound controls, in *D. virilis*, AttB was induced 2.9-fold (*t*(6.79) = –2.22, *p* = .063), while in *D. neotestacea* AttB was induced by 4.2-fold (*t*(13.25) = –4.99, *p* < 0.0005). Drosocin and diptericin in both *D. virilis* and *D. neotestacea* were not strongly induced by sterile wounding (*p* > 0.1). The *D. neotestacea* DptC was not upregulated by *Serratia* challenge, even relative to unwounded control flies. Drosocin is not appreciably induced by sterile wounding in either species, despite drosocin being induced by sterile wounding in *D. melanogaster* (Lemaitre et al., 1997); this difference in expression may be due to drosocin’s shift in genomic position between these two lineages (Fig. 3b). (PDF 439 kb)

## Abbreviations

Adh: Alcohol dehydrogenase
AIC: Akaike information criterion
Amd: Alpha methyldopa-resistant
AMP: Antimicrobial peptide
AttB: Attacin B
Bom: Bomanin
BSR: Branch-site REL
Dpt: Diptericin
DSCAM1: *Drosophila* down syndrome cell adhesion molecule
Gr43a: Gustatory receptor 43a
HMM: Hidden Markov model
IM: Immune-induced molecule
Imd: Immune-deficiency
JAK-STAT: Janus kinase- Signal-transducer and activator of transcription protein
JNK: Jun kinase
LRT: Likelihood ratio tests
ML: Maximum likelihood
Mtk: Metchnikowin
PGRP: Peptidoglycan recognition protein
REL: Random-effects likelihood
RpL: Ribosomal protein L
