# Supplementary figures and images for: Immune genes and divergent antimicrobial peptides in flies of the subgenus Drosophila
Source: BMC Evol Biol. 2016 Oct 24;16:228. doi: 10.1186/s12862-016-0805-y (PMC5078906; doi:10.1186/s12862-016-0805-y)

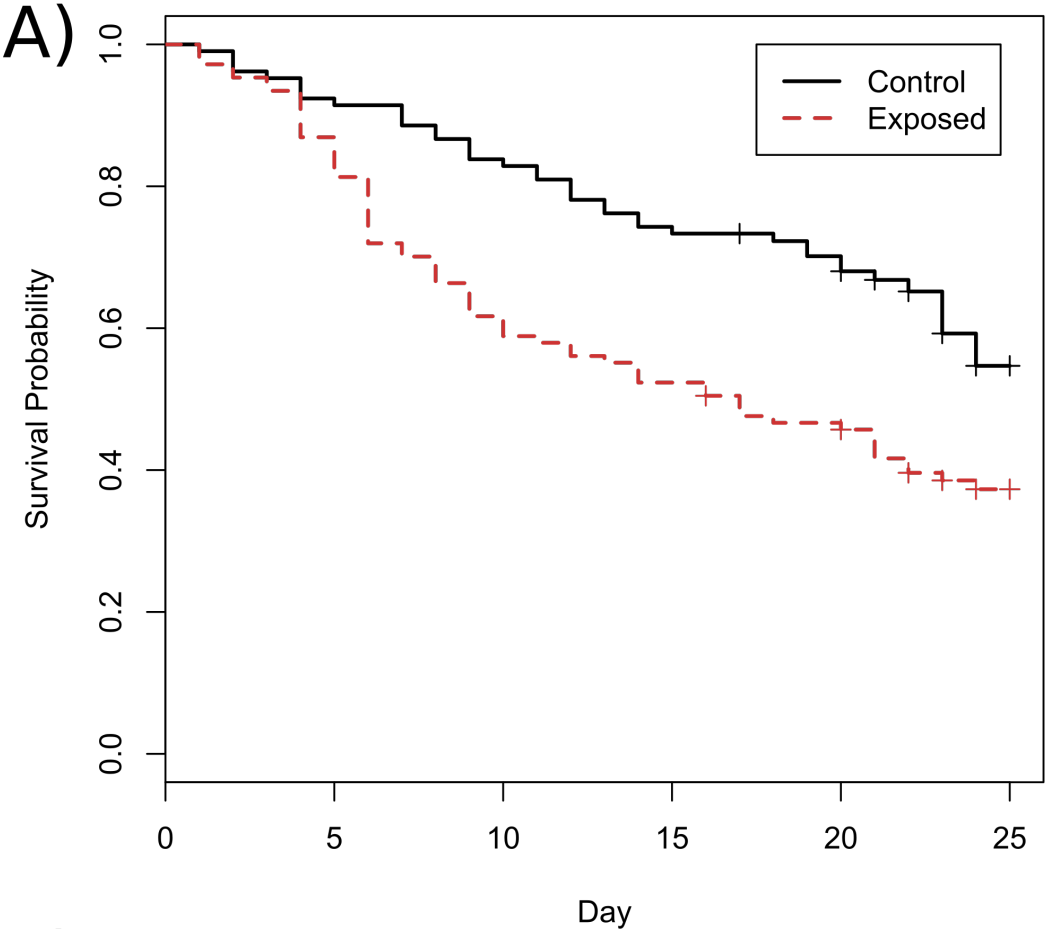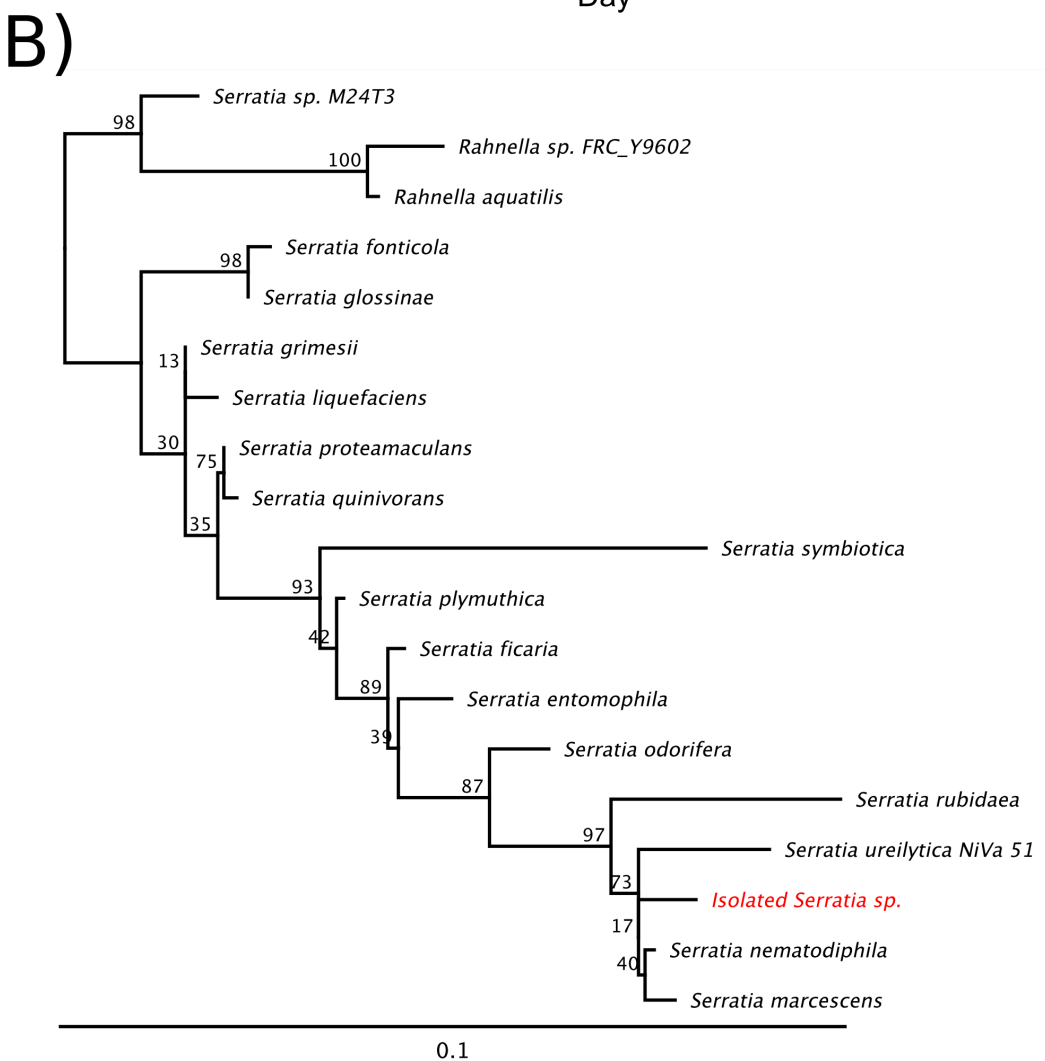

Supplement: Additional file 2: Figure S4. — Our Serratia strain is an oral pathogen of D. neotestacea related to Serratia marcescens. A) Flies were fed on mushroom agar with either 100uL of Luria-Bertani Broth (Control) or OD600 = 0.1 Serratia solution (Exposed) for 6 h, prior to transfer to sterile mushroom agar vials. Flies were turned over into new agar vials every 4 days, and mortality was recorded daily. Crosses indicate flies that were lost unrelated to treatment. Flies exposed to Serratia suffered significantly shorter lifespans compared to control treatments (n = 212; LR test: χ 2 = 11.8, p = 5.90e–4; GW test: χ 2 = 13.3, p = 2.66e–4). B) Maximum likelihood tree (100 bootstraps) of the isolated Serratia sp. 16S gene highlighted in red, with Rahnella sp. included as an outgroup. (PDF 390 kb) [file 12862_2016_805_MOESM2_ESM.pdf]

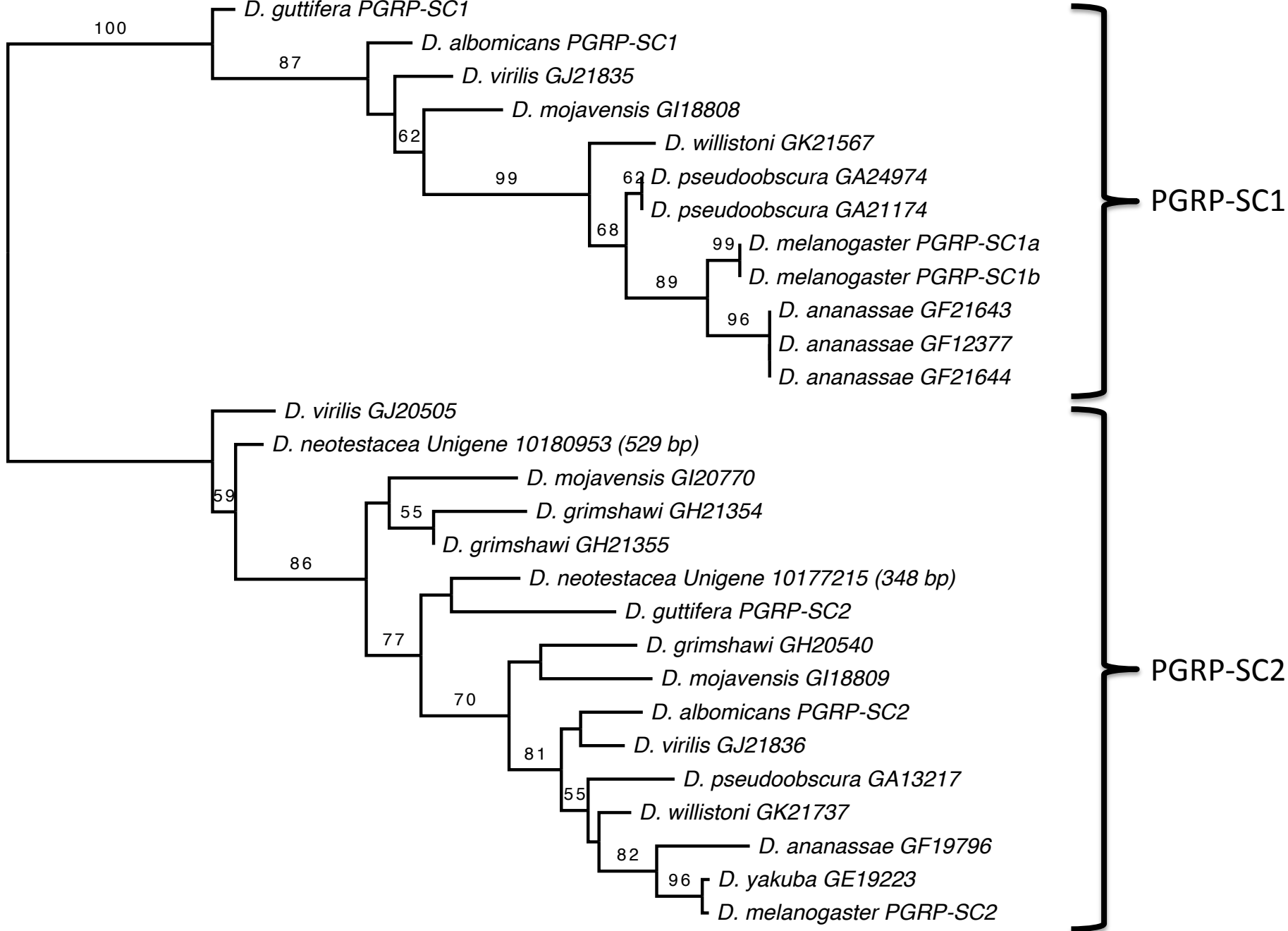

Supplement: Additional file 3: Figure S1. — Phylogenetic analysis of PGRP-SC1 and SC2 amino acid sequences using maximum likelihood. Support values indicate 100 bootstraps. Drosophila neotestacea has two PGRP-SC genes. The D. albomicans PGRP-SC1 signal peptide sequence is unresolved, and the current scaffold assembly in the D. guttifera genome does not contain the anterior region of its PGRP-SC1 orthologue. (PDF 45 kb) [file 12862_2016_805_MOESM3_ESM.pdf]

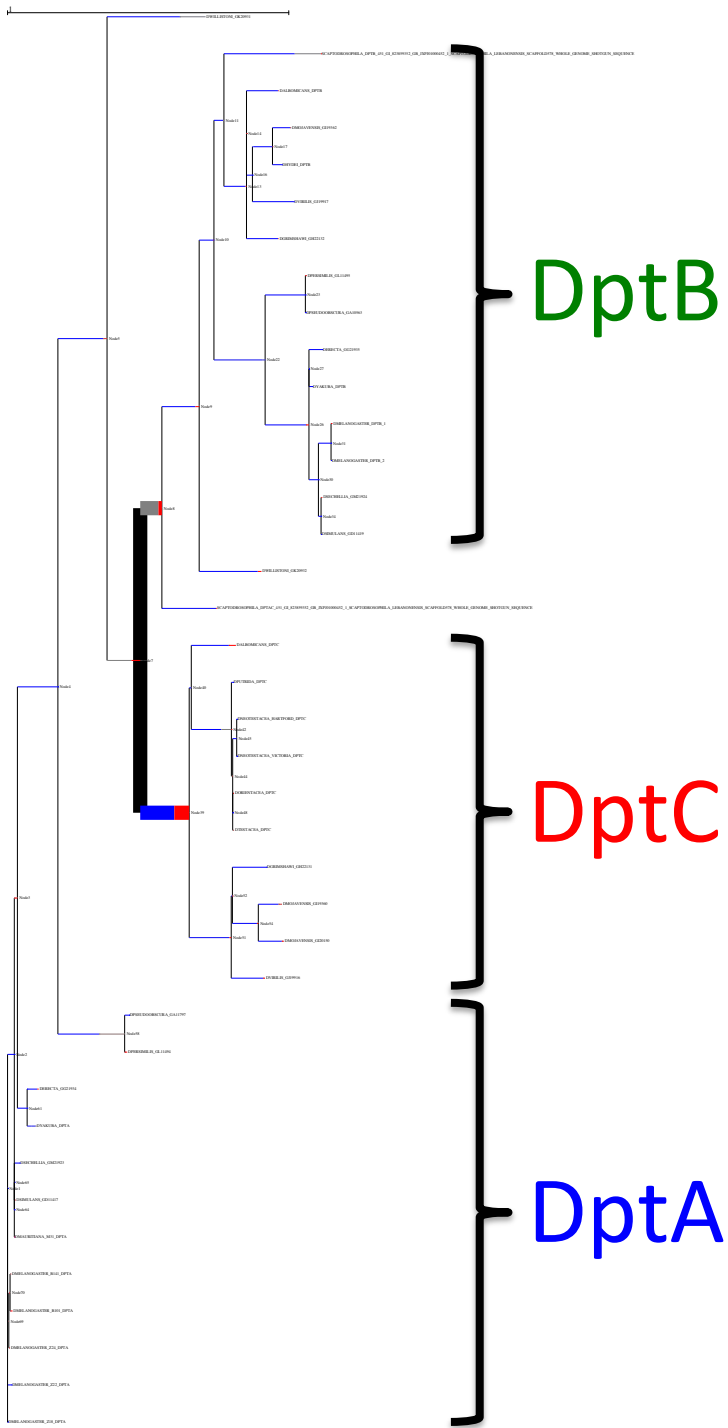

Supplement: Additional file 4: Figure S2. — Example branch-site REL analysis of Drosophila diptericins. Branch colour indicates the strength of selection, with red corresponding to dN/dS > 5, grey to dN/dS = 1, and blue to dN/dS = 0. The width of the colour on each branch represents the proportion of sites in the corresponding class. Bolded branches indicate branches that evolved under positive selection. In this BSR analysis, there is strong evidence that the root branch of the DptC clade diverged through positive selection (p < 0.001); there is also some evidence for positive selection at the divergence of DptA from DptB (p = .017). The codon alignment used in this analysis does not include D. ananassae diptericins, as D. ananassae DptA clustered with D. willistoni DptA on extremely long branches. This D. ananassae diptericin amino acid sequence can be seen in Fig. 4d, and consequences on net charge of D. ananassae DptA are shown in Additional file 5: Table S2. (PDF 47 kb) [file 12862_2016_805_MOESM4_ESM.pdf]

A)

Attacin B

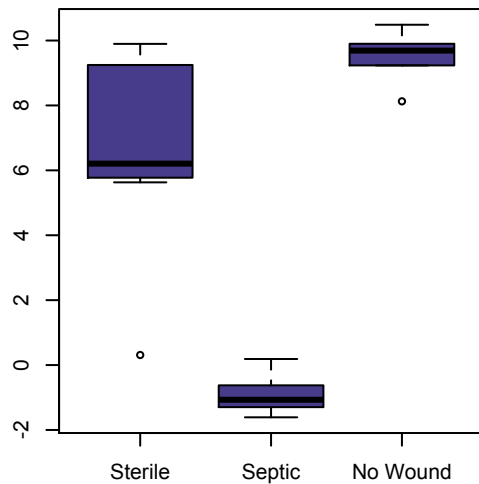

Drosocin

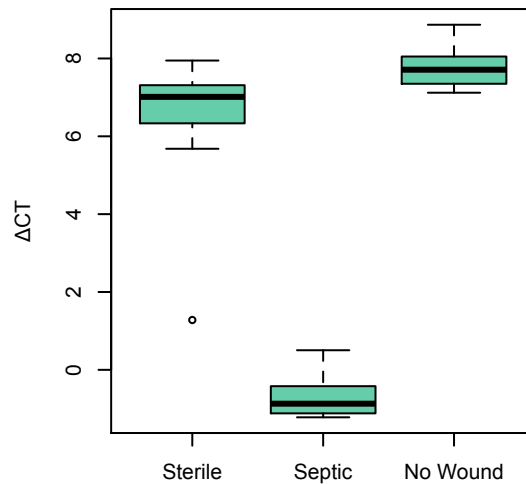

Diptericin C

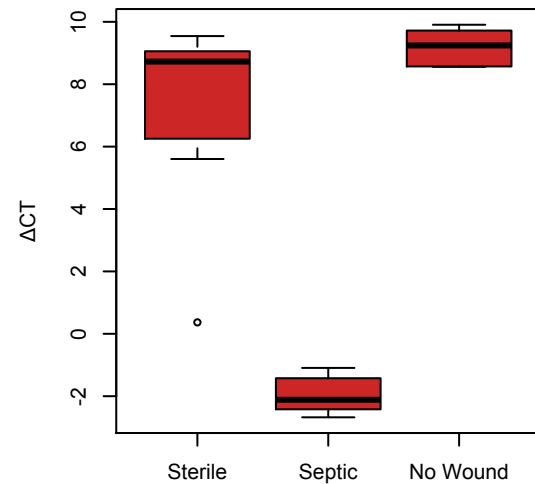

B)

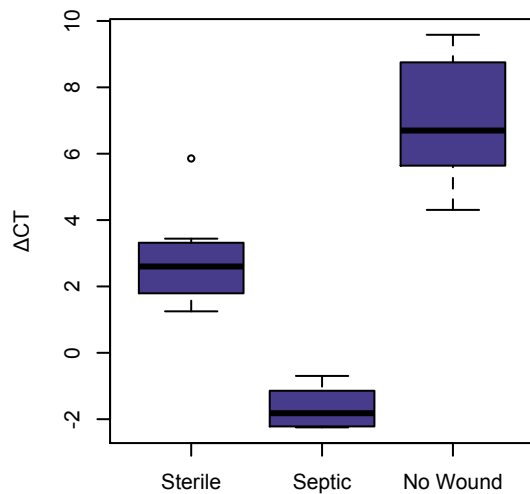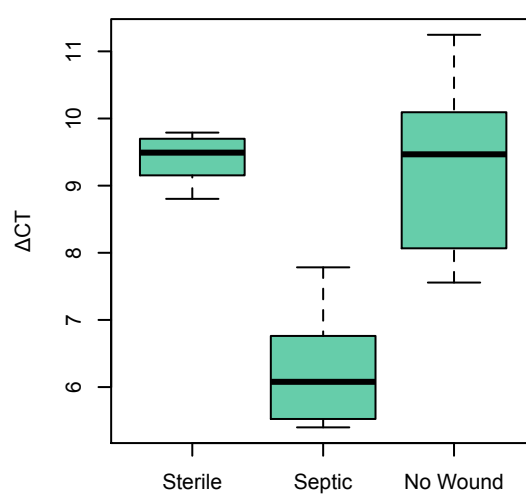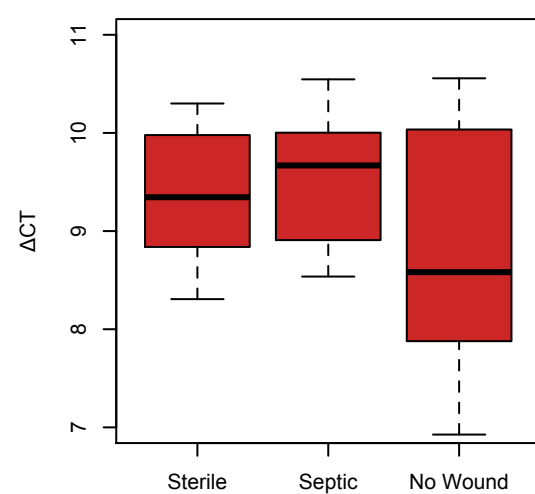

Supplement: Additional file 6: Figure S3. — AMP gene expression following no-wound controls in (A) D. virilis and (B) D. neotestacea. Treatments involved a sterile wound (Sterile), Serratia bacterial challenge (Septic), or a no-wound control (No Wound). Comparing sterile wound treatments to no-wound controls, in D. virilis, AttB was induced 2.9-fold (t(6.79) = –2.22, p = .063), while in D. neotestacea AttB was induced by 4.2-fold (t(13.25) = –4.99, p < 0.0005). Drosocin and diptericin in both D. virilis and D. neotestacea were not strongly induced by sterile wounding (p > 0.1). The D. neotestacea DptC was not upregulated by Serratia challenge, even relative to unwounded control flies. Drosocin is not appreciably induced by sterile wounding in either species, despite drosocin being induced by sterile wounding in D. melanogaster (Lemaitre et al., 1997); this difference in expression may be due to drosocin’s shift in genomic position between these two lineages (Fig. 3b). (PDF 439 kb) [file 12862_2016_805_MOESM6_ESM.pdf]
